# A Prospective Comparison of Bipolar I and II Subjects With and Without Comorbid Alcohol Dependence From the COGA Dataset

**DOI:** 10.3389/fpsyt.2020.522228

**Published:** 2020-12-21

**Authors:** Ulrich W. Preuss, M. N. Hesselbrock, V. M. Hesselbrock

**Affiliations:** ^1^Vitos Hospital Psychiatry and Psychotherapy, Herborn, Germany; ^2^Department of Psychiatry, Psychotherapy and Psychosomatics, Martin Luther-University Halle-Wittenberg, Halle, Germany; ^3^Department of Psychiatry, University of Connecticut School of Medicine, Farmington, CT, United States

**Keywords:** alcohol dependence, bipolar disorder, 5-year follow-up, comorbidity, suicidal behavior

## Abstract

**Objective:** Comorbidity of alcohol use disorders in bipolar subjects is high as indicated by epidemiological and clinical studies. Though a more severe course of bipolar disorder in subjects with comorbid alcohol dependence has been reported, fewer studies considered the longitudinal course of alcohol dependence in bipolar subjects and the prospective course of comorbid bipolar II subjects. Beside baseline analysis, longitudinal data of the COGA (Collaborative Study on Genetics in Alcoholism) were used to evaluate the course of bipolar I and II disordered subjects with and without comorbid alcohol dependence over more than 5 years of follow-up.

**Methods:** Characteristics of bipolar disorder, alcohol dependence and comorbid psychiatric disorders were assessed using semi-structured interviews (SSAGA) at baseline and at a 5-year follow-up. Two hundred twenty-eight bipolar I and II patients were subdivided into groups with and without comorbid alcohol dependence.

**Results:** Of the 152 bipolar I and 76 bipolar II patients, 172 (75, 4%) had a comorbid diagnosis of alcohol dependence. Bipolar I patients with alcohol dependence, in particular women, had a more severe course of bipolar disorder, worse social functioning and more suicidal behavior than all other groups of subjects during the 5-year follow-up. In contrast, alcohol dependence improved significantly in both comorbid bipolar I and II individuals during this time.

**Conclusions:** A 5-year prospective evaluation of bipolar patients with and without alcohol dependence confirmed previous investigations suggesting a more severe course of bipolar disorder in comorbid bipolar I individuals, whereas bipolar II individuals were less severely impaired by comorbid alcohol use disorder. While severity of alcohol dependence improved during this time in comorbid alcohol-dependent bipolar I patients, the unfavorable outcome for these individuals might be due to the higher comorbidity with personality and other substance use disorders which, together with alcohol dependence, eventually lead to poorer symptomatic and functional clinical outcomes.

## Introduction

There are converging lines of evidence demonstrating that substance use disorders are overrepresented in individuals with bipolar disorder. Compared to patients with other types of psychiatric conditions, including schizophrenia, panic disorder, major depression, and dysthymia, subjects with bipolar I and bipolar II disorder were reported to have the highest lifetime rates of alcohol or substance use disorders (ASUD) (1). The ECA Study reported a 60.7% lifetime prevalence rate for substance use disorders in persons with bipolar I disorder; alcohol was the most common substance abused (1). Individuals with bipolar I and bipolar II disorders had the highest lifetime prevalence rate of alcohol abuse or dependence (46.2 and 39.2%, respectively).

Several subsequent studies in epidemiological and clinical samples reported the rate of ASUD comorbidity in bipolar I and II patients to range between 14 and 65% (1, 2) compared to 6–12% in the general population (3).

A more recent epidemiologic investigation, the National Epidemiologic Survey on Alcohol and Related Conditions (NESARC) of more than 43,000 respondents reported a rate of 23.6% of any alcohol use disorders in bipolar I subjects during the last 12 months and over 56% during lifetime (4).

ASUD which co-occur with bipolar disorder were repeatedly reported to complicate the course of illness and lead to increased violence and hospitalization, greater suicide risk and poorer outcomes [reviewed by (5–8)].

Several studies indicate that bipolar subjects with comorbid ASUD have an increased rate of treatment non-adherence (9, 10). The reported consequences are less medication compliance (11), less symptomatic and functional recovery from affective symptoms (12), more aggressive and violent behaviors (7), higher rates of hospitalization and more often complications of bipolar disorders like rapid cycling and mixed episodes (6, 13–15).

These findings are supported by prospective studies which reassessed patients prospectively after initial admission for bipolar disorder (16). Over time, a similar profile of complications was reported for comorbid alcohol-dependent bipolar subjects as found in cross-sectional investigations including less treatment compliance (13, 14, 16) and worse socioeconomic, functional and symptomatic outcome (2, 16), while other studies did not report differences in number of days affected by mood symptoms, severity of mood episodes and social functioning across groups of abstinent, moderate and severe alcohol using BP I and II subjects (17).

In addition to ASUD, there is a high rate of other comorbidity with mental disorders in bipolar I and II subjects, including personality disorders, anxiety disorders and suicidal behavior (8, 10).

While most studies investigated characteristics of comorbid bipolar and ASUD subjects retrospectively, only a few investigations used prospective data to evaluate the course of both disorders over time by differentiating between bipolar I and II subgroups [review by Rakofsky and Dunlop (10)]. Since there are only a few prospective studies on the clinical course and prognosis of bipolar I and II patients with and without alcohol dependence, the COGA sample (Collaborative study on Genetics in alcoholism) was used to compare these two subgroups of bipolar individuals with and without comorbid alcohol dependence directly. The patients were assessed regarding their lifetime history of ASUD, bipolar disorders and then prospectively re-interviewed after 5 years. In these analyses, both bipolar 1 and II subjects are included to investigate first, the lifetime characteristics of alcohol dependence and bipolar disorders retrospectively and second, the course of alcohol dependence, bipolar disorder and comorbidity with mental and other substance use disorders during a 5-year follow-up period.

## Methods

The Collaborative Study on the Genetics of Alcoholism is a family pedigree investigation which enrolls treatment-seeking alcohol-dependent probands who meet the DSM-IV for alcohol dependence (18). Probands are recruited at six centers in the United States. The only exclusions are for life-threatening medical disorders, repeated intravenous drug use, and an inability to speak English. Comparison subjects have been identified from driver's license records, attendance at a dental clinic, random mailings, and other approaches (19). Written informed consent to participate in the study was obtained from all enrolees. Probands and their relatives were interviewed at baseline by using the Semi-Structured Assessment for the Genetics of Alcoholism (SSAGA), which focuses on demography, substance use patterns, and the assessment of 17 axis I DSM-IV diagnoses, as well as characteristics of bipolar disorder (20).

Though the SSAGA was developed prior to the publication of the DSM-IV criteria, all criteria symptoms for the DSM-IV diagnosis were queried, as well as times of onset and remission of symptoms (19). Comparison subjects were interviewed in the same format at baseline (wave I, re-evaluation wave II after 5 years). Only original probands or comparison subjects, their first-degree relatives, and offspring age ≤20 years in the participating families were eligible for follow-up. Of eligible subjects, the follow-up rate was 60% in probands, 65% in family members, and 78% in controls (19).

The interviewer also asked about past affective disorders, including depressive and manic episodes and the characteristics of the most severe episode. If subjects reported a lifetime diagnosis of both DSM IV major depression and mania or any lifetime diagnosis of a manic episode, they were affected by bipolar I disorder, while those persons who had at least one major depression and hypomanic episodes were diagnosed to have bipolar II disorder. Altogether, 201 subjects with bipolar I or II disorder were identified using these criteria of whom 152 (75.6%) had an additional diagnosis of DSM IV alcohol dependence (56 of 76, 74%, in bipolar I subjects and 96 of 125, 77%, in bipolar II subjects). Subjects with a bipolar II disorder without comorbid alcohol dependence were included into group 1 (*n* = 20) while a second group (group 2, *n* =56) encompassed those with a comorbid bipolar II and alcohol dependence diagnoses. Group 3 included subjects with a bipolar I diagnosis without alcoholism (*n* = 29) and group 4 consists of bipolar I subjects with a comorbid alcohol dependence (*n* = 96). Individuals with a history of mania (*n* = 27) without depression at baseline were excluded from the subsequent analyses.

The probands, comparison subjects, and appropriate relatives were re-assessed at a mean of 5, 72 years (SD = 1.1) after the initial interview.

The SSAGA interview provides several questions regarding current psychopathology including affective symptoms. The instruction to the interviewer was “All items on this rating sheet refer to behavior, attitudes, cognition, and appearance during the Interview.” Items were answered “yes” or “no.” (1 or 0). For affective symptoms The psychopathological Items included at both wave I and II affective symptoms regarding facial expression (e.g., sad, gloomy, hostile…), psychomotor activity (e.g., agitation, tics, tremor…), flow of thoughts (e.g., blocking, circumstantial, tangential…), mood and affect (e.g., depressed mood, labile mood, elated mood…, 6 items), content of thought (e.g., Ideas of hopelessness, Ideas of worthlessness, delusions of grandeur…) and others. All individuals included into the analyses had an average baseline symptom count (range 0–6) of 0.21 ± 0.65 and 0.49 ± 0.84 at follow-up. The rate of individuals who had more than 2 mood symptoms was *n* = 11 (5.5%) at baseline and *n* = 28 (13.9%) at follow – up. Most of the participants were in an euthymic mood state at the time of the interview.

Of the 228 bipolar subjects interviewed at baseline, 121 (53.1%) subjects were successfully re-evaluated at follow-up using the SSAGA (Group 1: 65.0%, *n* = 13; Group 2: 53.6%; *n* = 30; Group 3: 58.6%, *n* = 17; group 4: 63.5%; *n* = 61). There were no significant differences across groups regarding the rate of persons re-interviewed, nor were there differences across groups regarding age, gender and symptoms during the most severe depressive or manic episode when re-evaluated subjects were compared to those who could not be re-assessed at follow-up. All other individuals did not agree to be re-interviewed, deceased (*n* = 2) or could not be located due to address change.

All SSAGA interviewers were instructed to evaluate whether the study participant is in euthymic mood or affective state at the time of the SSAGA assessment. All individuals were interviewed in an outpatient setting usually located in a research lab interview room. All the participants were in euthymic state at time of the interview.

Additional sections of the SSAGA were used to determine the age at onset of substance use disorders (i.e., the age by which three or more criteria were met) and of further psychiatric conditions like DSM IV anxiety disorders, antisocial personality and conduct disorder.

Psychopathology and behavior during the interview observed by the interviewer included assessments of appearance, orientation, level of consciousness, memory, mood and formal thought. Global level of functioning was obtained using the GAF (18) at baseline and at the follow-up interview.

Differences across groups were evaluated by using chi-square tests for categorical data and one-way analysis of variance (ANOVA). Scheffé *post-hoc* tests were used to determine significant differences in specific group comparisons. To compare characteristics of continuous variables over time, repeated measurement ANOVA (MANOVA) was used. For *post-hoc* group comparisons, Scheffé *post-hoc* tests were employed.

## Results

### Sample Characteristics

Sociodemographic characteristics of the four groups are presented in Table 1. At the baseline interview, subjects with non-comorbid bipolar II disorder (Group 1) were significantly younger than those with bipolar I disorder alone (Group 3). While no further differences across groups were detected regarding years of education, gender, ethnicity, marital status and holding of college degrees, a significantly higher proportion of group 4 members compared to group 1 were unemployed.

**Table 1:** Baseline Demographic Characteristics of Bipolar 1 and 2 Subjects Divided by Alcohol Dependence Diagnosis.

**Alc dep diagnosis (DSM IV) variables**	**Group 1 (*n* = 20) BP2+ Alc-**	**Group 2 (*n* = 56) BP2+ Alc+**	**Group 3 (*n* = 36) BP1+ Alc-**	**Group 4 (*n* = 116) BP1+ Alc+**	**Group comparisons *F* or χ^**2**^ value**	**Group comparisons**
**Mean (± SD)**						
Age (years) Baseline	29.75 ± 11.6	36.36 ± 9.6	39.61 ± 12.9	36.51 ± 9.1	4.08^*^	1 vs. 3
**Current age (years)**						
Years of education	13.00 ± 1.9	12.21 ± 2.0	12.50 ± 2.7	12.60 ± 2.0	0.76	
**Categorical variables (%)**						
Female gender	70.0%	42.9%	69.4%	48.3%	9.50^*^	1.3 vs. 4
Ethnicity:					6.37	
Caucasian Afr. Am. Hispanic Other	80.0% 10.0% 5.0% 5.0%	80.4% 17.9% 0.0% 1.8%	69.4% 19.4% 8.3% 7.0%	72.2% 16.5% 7.0% 4.3%		
Marital Status:					12.43^**^	1.3 vs. 4
Married Widowed Separat./Divor. Never Marr.	45.0% 0.0% 15.0% 40.0%	37.5% 0.0% 25.0% 37.5%	52.8% 0.0% 22.2% 25.0%	33.0% 2.6% 36.5% 27.8%		
Unemployed	25.0%	35.7%	38.9%	58.3%	13.55^**^	1.2 vs. 4
College degree	15.0%	3.6%	13.9%	10.4%	3.83	

* *p < 0.05*;

** *p < 0.01*.

### Baseline Analyses

Regarding characteristics of alcohol dependence, individuals with bipolar I and II with alcohol dependence were compared. The only difference found between groups were a higher rate of alcohol-related liver disease in group 2 subjects compared to group 4 members (Table 2).

**Table 2:** Baseline Alcohol dependence-related characteristics and treatment histories of Bipolar 1 and 2 Subjects Divided by Alcohol Dependence Diagnosis.

**Alcohol Dependence characteristics (DSM IV) Variables**	**Group 1 (*n* = 20) BP2+ Alc-**	**Group 2 (*n* = 56) BP2+ Alc+**	**Group 3 (*n* = 36) BP1+ Alc-**	**Group 4 (*n* = 116) BP1+ Alc+**	**Group comp.: T or *χ^2^* value**	**Single group comparisons**
**Group 2 vs. 4, Mean (± SD)**						
First age of regular drinking		18.07 ± 6.0		17.49 ± 5,9	0.59	
Age of onset of alc. dep.		21.95 ± 7.6		21.65 ± 7.7	0.23	
Max. number drinks per day		34.89 ± 28.3		35.70 ± 32.0	−0.16	
Number of DSM-IV criteria endorsed		5.79 ± 1.16		5.86 ± 1.51	−0.31	
Number of withdrawal symptoms		3.96 ± 3.1		4.53 ± 3.19	−1.01	
Number or alc-rel. violence		2.71 ± 1.6		2.94 ± 1.4	−0.93	
Number of alc.-rel. physical problems		0.75 ± 1.0		0.76 ± 1.4	1.75	
**Categorical variables (%)**						
**Alcoholism treatment (Group 2 vs. 4 only)**						
Inpatient treatment		51.8%		70.4%	3.31	
Outpatient treatment		28.6%		47.8%	5.70^*^	2 vs. 4
Alcoholics anonymous		62.5%		69.6%	0.85	
**Alcohol-related violence**						
Arguments		31.7%		57.9%	0.93	
Throw objects		20.9%		49.7%	2.34	
Hit family members		11.7%		28.1%	0.57	
Hit others		11.7%		20.5%	0.48	
Physical fights		18.7%		41.5%	0.33	
**Alcohol-related physical problems**						
Liver disease		10.7%		1.7%	6.80^**^	2 vs. 4
Stomach disease		23.2%		13.0%	2.84	
Pancreatitis		3.6%		4.3%	0.06	
Heart disease		0.0%		1.7%	0.98	
PNP		25.0%		14.8%	2.64	
Memory problems		58.5%		62.2%	0.21	
**Other mental comor-bidity (Groups 1–4):**						
Panic disorder	5.0%	17.9%	16.7%	24.1%	4.49	
Agoraphobia	10.0%	8.9%	16.7%	16.4%	2.21	
Conduct disorder	5.0%	37.5%	11.1%	32.9%	14.21^**^	1.3 vs. 2.4
Antisocial personality	5.0%	30.4%	5.8%	29.3%	13.82^**^	1.3 vs. 2.4

* *p < 0.05*;

** *p < 0.01*.

While there was no other difference between groups 2 and 4 individuals, members of both groups were more likely to be dependent on illicit substances (results not shown), including marihuana, cocaine, amphetamines, sedatives or hypnotics and opiates, and more often reported a lifetime history of conduct and antisocial behavior (Table 3).

**Table 3:** Baseline Characteristics of Bipolar Disorders of Bipolar 1 and 2 Subjects Divided by Alcohol Dependence Diagnosis.

**Affective disorder characteristics (DSM IV) variables**	**Group 1 (*n* = 20) BP2+ Alc-**	**Group 2 (*n* = 56) BP2+ Alc+**	**Group 3 (*n* = 36) BP1+ Alc-**	**Group 4 (*n* = 116) BP1+ Alc+**	**Group comp.; F, T or χ^**2**^ value**	**Group comparisons**
**Mania (bipolar I)/hypomania (bipolar II)**						
Mania/hypomania age of onset	25.45 ± 11.6	31.18 ± 10.2	32.25 ± 12.3	31.54 ± 9.8	2.50	
Treatment most severe manic/hypomanic episode Medication Hospitalization ECT	10.5% 100.0% 0.0% 0.0%	16.1% 60.0% 0.0% 0.0%	52.8% 68.4% 31.6% 5.3%	48.3% 71.4% 35.7% 1.8%	26.08^***^	1.2 vs. 3.4
# symptoms most severe manic/hypomanic episode	4.26 ± 1.0	5.13 ± 1.7	6.36 ± 2.0	7.07 ± 1.9	22.16^***^	2.3 vs. 4
**Depression**						
# depressive episodes	4.75 ± 4.0	14.61 ± 24.2	7.79 ± 11.6	13.62 ± 22.2	0.84	
Age of onset depression	21.75 ± 10.1	19.73 ± 9.6	24.53 ± 10.4	21.14 ± 10.5	1.52	
Any professional treatment most severe episode Medication Hospitalization ECT	40.0% 75.0% 25.0% 0.0%	44.6% 60.0% 24.0% 0.0%	68.8% 59.1% 27.3% 4.5%	51.8% 68.4% 45.6% 10.5%	11.17^*^	
# symptoms most severe depressive episode	7.46 ± 2.0	9.12 ± 2.1	8.65 ± 1.9	8.91 ± 1.8	2.57	
Affective symptoms at baseline interview (Interviewer rating)	0.10 ± 0.4	0.14 ± 0.4	0.19 ± 0.7	0.30 ± 0.8	1.10	
GAF baseline (Interviewer rating)	75.40 ± 12.3	66.46 ± 11.8	68.33 ± 9.8	58.22 ± 13.4	15.94^***^	1, 2, 3 vs. 4
**Other characteristics**						
Any mixed episodes	42.1%	28.6%	36.1%	39.1%	2.12	
Any rapid cycling	52.6%	58.9%	52.8%	67.2%	3.56	
Suicide ideation	80.0%	82.1%	77.8%	77.4%	0.55	
Suicide attempts	30.0%	48.2%	27.8%	38.8%	4.58	
# suicide attempts	1.67 ± 1.2	5.85 ± 18.7	1.40 ± 0.5	4.57 ± 9.6	0.41	
Age first suicide attempt	19.50 ± 10.4	26.93 ± 10.2	25.20 ± 8.4	28.14 ± 9.1	1.56	

* *p < 0.05*;

*** *p < 0.001*.

Comparing characteristics of mania across groups, bipolar I groups 3 and 4 individuals, not surprisingly, received treatment significantly more often during their manic episodes, were more often hospitalized and had a higher number of symptoms during their most severe episode compared to bipolar II groups 1 or 2 subjects. While there was no difference between groups 2 and 4 in number of symptoms during their most severe depressive episode, individuals of these two groups had significantly more symptoms than group 1 members. No differences were found for ages at onset of mania or depression and rates of subjects receiving medication or electroconvulsive therapy (ECT) in both mania and depression episodes across groups.

No significant differences across groups were obtained in rates of lifetime mixed episodes, rapid cycling and suicidal behavior, including suicidal ideation and history of suicide attempts, number of suicide attempts and age at onset of first suicide attempt, while highest values were found for group 4 individuals.

### Prospective Analyses

Characteristics of alcohol and substance consumption were compared again in groups 2 and 4. Remarkably, three individuals with alcohol dependence which were assigned into the bipolar II group at baseline, developed a manic episode during follow up and changed diagnosis to bipolar I with alcohol dependence.

All groups were included into the comparison of other mental disorders comorbidity during the 5-year follow-up. The results are presented in Table 4. Regarding characteristics of alcohol dependence and associated social and behavioral consequences, the only difference between the 2 groups during the follow-up period was a higher rate of binge drinking in alcohol-dependent bipolar I subjects. Including all groups into the analysis of drug-related characteristics during follow-up, group 4 members reported significantly more often withdrawal symptoms from cocaine and a higher frequency of drug-related treatment than all the other groups.

**Table 4:** Characteristics of Alcohol and Substance Dependence during 5 year Follow-Up in Bipolar 1 and 2 Subjects Divided by Alcohol Dependence Diagnosis.

**Alcohol dependence characteristics (DSM IV) variables**	**Group 1 (*n* = 13) BP2+ Alc-**	**Group 2 (*n* = 30) BP2+ Alc+**	**Group 3 (*n* = 17) BP1+ Alc-**	**Group 4 (*n* = 61) BP1+ Alc+**	**Group comp.; *F*, or χ^**2**^ value**	**Group comparisons**
**Comparison of group 2 vs. 4**						
**Alcohol-related behavior during follow-up period**						
Binge drinking		13.3%		31.1%	4.14^*^	2 vs. 4
Craving for alc.		6.7%		26.2%	3.14	
Desire to cut down		40.0%		44.3%	0.83	
Drunk larger amounts		61.1%		80.6%	2.36	
Tolerance		23.3%		18.0%	0.04	
Alcohol withdrawal		13.3%		13.1%	0.02	
Withdrawal seizures		0.0%		4.9%	1.79	
Delirium tremens		6.7%		8.2%	0.01	
Blackouts		36.7%		44.3%	0.83	
Drink during serious illness		3.3%		6.6%	0.63	
Great deal getting alcohol		13.3%		27.9%	2.20	
Given up activities		30.0%		41.0%	1.34	
Arrested for drunk driving		13.3%		24.6%	1.39	
Arrested for drunk behavior		10.0%		24.6%	2.33	
Alcohol-related marital problems		23.3%		31.1%	1.33	
Alcohol-related fights		36.7%		55.7%	3.28	
Alcohol-related injury		10.0%		14.8%	0.23	
Treatment for AUD		33.3%		54.1%	3.73	
Alcohol-related mental problems		20.0%		39.3%	3.40	
**Illegal substance use and consequences (Groups 1–4)**						
Follow-up marihuana use	20.0%	21.4%	23.5%	50.8%	5.25	
Follow-up marihuana treatment	0.0%	5.4%	5.9%	9.8%	1.54	
Follow-up withdrawal cocaine	0.0%	0.0%	0.0%	7.3%	7.93^*^	1, 2, 3 vs. 4
Follow-up withdrawal stimulants	0.0%	0.0%	0.0%	3.1%	3.33	
Follow-up withdrawal sedatives	0.0%	1.8%	0.0%	4.2%	2.48	
Follow-up withdrawal opioids	0.0%	0.0%	0.0%	6.3%	6.76	
**Characteristics of psychiatric comorbidity and suicidal behavior during follow-up (Groups 1–4)**						
Follow-up depression	50.0%	28.6%	58.8%	67.2%	4.37	
Follow-up dysthymia	0.0%	10.7%	23.5%	19.7%	2.94	
Follow-up manic episode	0.0%	5.4%	23.5%	34.4%	11.20^*^	1, 2 vs. 4
Follow-up hypomanic episode	0.0%	1.8%	5.9%	3.3%	0.74	
Follow-up panic attacks	0.0%	8.9%	11.8%	19.7%	3.37	
Follow-up suicide attempt	0.0%	0.0%	0.0%	18.0%	12.73^**^	1, 2, 3 vs. 4
Follow-up suicidal ideations	10.0%	14.3%	29.4%	45.9%	11.90^**^	1, 2, 3 vs. 4
Affective symptoms at follow-up interview (Interviewer rating)	0.20 ± 0.6	0.39 ± 0.7	0.31 ± 0.7	0.65 ± 0.8	3.11^*^	1, 2, 3 vs. 4
GAF follow-up (Interviewer rating)	77.2 ± 12.8	62.0 ± 26.7	69.2 ± 19.6	59.8 ± 19.3	3.32^*^	1, 2, 3 vs. 4

* *p < 0.05*;

** *p < 0.01*.

Regarding comorbidity and related characteristics during the 5-year follow-up, group 4 members had significantly more often suicide attempts, suicidal ideation and, not surprisingly, a manic episode than groups 1 and 2 individuals. However, 3 individuals (of *n* = 30) with baseline bipolar II and comorbid alcohol dependence reported a manic episode during follow-up. Furthermore, subjects of group 4 attempted more often suicide compared to group 3 individuals.

### Medication, Functional, and Affective Syndrome Changes Over Time of Bipolar Groups

While there was no difference in rate of subjects receiving medication during their most severe affective episode across groups, subjects were prescribed antidepressants (31%), benzodiazepines (18%), neuroleptics (18%), lithium (9%) and anticonvulsants (13%) or a combination of these medications (14%). At the follow-up interview, again no difference in kind and rate of medication for treatment of the most severe affective episode across groups was found. Patients were prescribed antidepressants in 11%, benzodiazepines in 7%, neuroleptics in 11%, lithium in 32%, and anticonvulsants in 12% of the cases. Twenty seven percentage of the bipolar subjects in all groups took a combination of these compounds at follow-up.

Regarding affective symptoms which were rated by trained SSAGA interviewers, significant differences between bipolar groups were detected over time (MANOVA F-value: 4.04; df 3; *p*: 0.009). *Post-hoc* tests revealed that groups 1–3 individuals improved while group 4 individuals deteriorated regarding their magnitude of affective symptoms during the follow-up period. Regarding social functioning over time, GAF scores remained unchanged or improved in groups 1 to 3 subjects over time, while for group 4 subjects a significant decrease of their level of social functioning was observed (MANOVA *F*-value: 9.92; df 3; *p* < 0.001) (Tables 3, 4).

### Gender-Specific Analyses

Across the four groups, the majority of the subjects were females, except group 2. To investigate potential gender-specific differences between bipolar I and II subjects with and without comorbid alcohol dependence, the analyses were repeated separately for both sexes. Regarding the baseline assessment, while male subjects were more often unemployed and had a lower rate of college degrees, significantly more alcohol-related violence (including more arguments and physical fights), a higher number of alcohol-related problems (*T*-value: 1.99, *p* = 0.049) and antisocial personality disorder diagnoses (χ^2^ value: 10.59, *p* = 0.014). Females were more often treated for mania (χ^2^ value: 10.49, *p* = 0.015) and reported higher number of symptoms during the most severe depressive episode (*F*-value: 3.75, *p* = 0.014), whereas males more often received medication during their most severe mania (χ^2^ value: 16.66, *p* = 0.001). During the follow-up period, females reported a more often craving (χ^2^ value: 12.26, *p* = 0.007), alcohol-related violent incidents (χ^2^ value: 13.40, *p* = 0.004), mental problems (χ^2^ value: 9, 58, *p* = 0.02), groups 2 and 4.

In comparison, males tended to have more manic episodes (χ^2^ value: 7, 37, *p* = 0.06) and more suicide attempts (χ^2^ value: 6, 10, *p* = 0.09) during the 5-year period.

## Discussion

Course of bipolar I and II with and without alcohol dependence in this sample is remarkably similar when only baseline analysis is considered. In the prospective analysis, however, bipolar I subjects with comorbid alcohol dependence had a worse course of bipolar disorder. However, no worse characteristics of alcohol dependence were found when compared to bipolar II subjects with comorbid alcohol dependence. This group of comorbid individuals had more suicidal behavior, manic episodes, drug-related problems and affective symptoms during the follow-up period as well as deterioration in social functioning. Compared to group 4 individuals, significant improvements in most of these characteristics were found for all bipolar II subjects with and without alcohol dependence and in bipolar I subjects without alcohol dependence (group 3).

Regarding characteristics of alcohol dependence during the follow-up period, remarkably few differences were found in comorbid alcohol-dependent bipolar I and II subjects. With the exception of a higher rate of binge drinking in bipolar I subjects, both groups of bipolar subjects were equally affected by alcohol-related behavior and complications during the follow-up period.

A high comorbidity of bipolar disorders with alcohol dependence and the more severe course of bipolar I disorder in these comorbid individuals in this analyses correlate with results from several previous prospective studies of which several suggest that alcohol does increase the risk of a mood recurrence (12, 14, 16, 21, 22) while a minority group of investigations suggest the opposite (13, 17).

Also in accord with previous studies is the high rate of substance use disorders, suicidal behavior and other axis I disorders in these subjects (23–25). Furthermore, social functioning and depressive symptoms were worst in group 4 subjects, confirming previous results from both short-term (26–28) and prospective studies (14, 16). Moreover, previous investigations also reported that members of this group of individuals had a higher rate of manic episodes during follow-up (25, 29). Clearly, the more severe course of comorbid bipolar I disorder, and alcohol dependence was more pronounced in the prospective compared to the baseline analysis.

Course of alcohol dependence in bipolar I was very variable during follow-up. At least one criterion for alcohol dependence was present in 13–41% of the individuals of comorbid bipolar II subjects compared to bipolar I (13–80%). However, this result also indicates that the majority of bipolar I subjects who had comorbid alcohol dependence at baseline did improve significantly during this time.

In contrast to bipolar I patients, comorbid alcohol dependence may have little influence on the course of bipolar disorder in bipolar II individuals in the current sample. Except for severity of symptoms during their most severe depressive episodes, the course of bipolar II disorder is very similar in both groups with and without comorbid alcohol dependence. Furthermore, no significant differences were found in alcohol dependence and bipolar characteristics between the two groups of bipolar I and II subjects during the 5 years of follow-up were found. These results confirm the findings from the baseline analysis. While only few studies considered bipolar II subjects in their studies on comorbid alcohol use disorders in bipolar individuals, a previous investigation reported almost equal rates of alcohol dependence in bipolar II compared to bipolar I individuals (30). In comparison, a more recent prospective study found more than twice the rate of alcohol dependence in bipolar II vs. I patients (31), while other samples did not detect any influence of alcohol use on mood symptoms or social functioning in bipolar II individuals too (17).

Previous studies also repeatedly stated the hypothesis that alcohol consumption in bipolar patients is an attempt to counteract unpleasant affective symptoms. However, the findings from empirical investigations are not unanimous. For instance, a re-analysis of a prospective study on manic or mixed bipolar I individuals demonstrated no consistent pattern of temporal correlation between alcohol use disorders and affective symptoms (32).

In another study, 77 bipolar patients were prospectively investigated over 12 months to assess the associations between alcohol use and bipolar disorders after a first hospitalization of mania (33). Most alcohol-dependent bipolar subjects developed an additional affective episode during follow-up in the absence of ongoing alcohol or drug abuse. The authors concluded that patients whose bipolar illness co-occurs with alcohol or substance use disorders do not necessarily require ongoing alcohol or substance abuse to initiate new affective episodes. But when present, high alcohol use or use disorders were associated with affective symptoms. In the current analyses, while not statistically significant, the number of affective symptoms in the comorbid bipolar I group worsened during the follow-up.

Despite the inconsistent relationship of alcohol use and alcohol dependence characteristics with affective episodes over time, subjects with bipolar I disorder and alcohol dependence in the current sample do in general worse than bipolar II or non-comorbid bipolar I subjects. Another reason why this group of patients has a more severe course of the affective disorder might lie in their high rate of psychiatric comorbidity. Although a higher rate of anxiety disorders was not found in comorbid bipolar subjects, groups 2 and 4 individuals met significantly more criteria for conduct and antisocial personality disorders and experienced significantly more alcohol-related violence, in particular women. Women accounted for many of the significant differences across the 4 groups, including alcohol dependence severity, alcohol-related aggression and physical consequences and more severe alcohol use disorder-related characteristics, lower social functioning and a higher amount of affective symptoms during the follow-up period. These individuals must therefore have reached a remarkable severity of both disorders and might therefore represent a high-risk group within these comorbid individuals. A previous study reported that the risk of having an alcohol dependence was greater for women with bipolar disorder (odds ratio = 7.35) than for men with bipolar disorder (odds ratio = 2.77), compared with the general population (34).

While the course of alcohol dependence is improved during follow-up, its remaining symptoms still might interact with characteristics of psychiatric comorbidity and result in less treatment adherence and lower medication compliance. Together it might result in poorer symptomatic and functional recovery of these highly comorbid bipolar subjects (14, 33). Previous studies also noted that comorbid psychiatric disorders exacerbate bipolar disorder even without the negative effects of alcohol or substance abuse (30, 35).

Of course, there are several limitations to this analysis. First, primarily alcohol-dependent subjects in treatment, their relatives and control families were enrolled into this study. This might explain the rather high rate of alcohol dependence diagnosis of more than three quarters in this bipolar sample compared to previous studies. Second, several previous investigations included first-episode manic patients to overcome potential bias caused by the number of affective episodes and chronic course of bipolar disorders. The COGA sample enrolled subjects with and without alcohol dependence; its target group was not first-episode bipolar subjects. Thus, more chronic bipolar disorder individuals who were currently not in treatment were recruited. Chronic patients have a higher rate of previous affective episodes which in turn increase the likelihood of future affective episodes. When subjects at different stages of their disease are investigated, it is more difficult to identify other course predictors (33) and to evaluate the influence of a comorbid disorder on prognosis of bipolar disorder.

The sample is not from a clinical population but rather a family study. Comparison of the study's results to inpatient samples is therefore limited. Not all potential comorbidity (e.g., all personality disorders) were assessed in this family study. However, the structured clinical interview employed (SSAGA) covered many relevant comorbid mental disorders and characteristics of alcohol and substance use disorders in bipolar individuals.

Several additional characteristics of bipolar disorder, including rapid cycling and mixed episode were not traced during the follow-up period. Thus, a higher rate of these potential complications of bipolar disorder in comorbid subjects could not be investigated prospectively.

A significant number of subjects could not be re-interviewed after 5 years, mainly because their address has changed and could not be contacted (~50%). However, characteristics of re-interviewed and not-re-interviewed individuals did not differ significantly across groups.

Finally, contrasting results of baseline and follow-up analyses are often observed and might be due to a difference in patient's recall when asked for lifetime events in comparison to events during a shorter and more recent period of time. In the COGA study, subjects were assessed at baseline regarding their current and lifetime characteristics. At the follow-up interview, they were again assessed for their current and lifetime characteristics and in addition, for most recent events during this period. Thus, individuals might recall these more recent events better than more remote features of their disorders. This also underscores the necessity for prospective studies which might provide more accurate data than solely baseline designs to reduce individual recall bias.

## Data Availability Statement

The datasets generated for this study are available on request to the corresponding author.

## Ethics Statement

The studies involving human participants were reviewed and approved by NIAAA ethics committee. The patients/participants provided their written informed consent to participate in this study.

## Author Contributions

VH and MH were part of the COGA Study. UP contributed to data analysis and writing of the manuscript. All authors contributed to the article and approved the submitted version.

## Conflict of Interest

UP received speaker honoraria from Johnson & Johnson during the last 3 years. The remaining authors declare that the research was conducted in the absence of any commercial or financial relationships that could be construed as a potential conflict of interest.
